# The Impact of Gene Polymorphisms on the Success of Anticholinergic Treatment in Children with Overactive Bladder

**DOI:** 10.1155/2015/732686

**Published:** 2015-06-24

**Authors:** Serhat Gurocak, Ece Konac, Iyimser Ure, Cem Senol, Ilke Hacer Onen, Sinan Sozen, Adnan Menevse

**Affiliations:** ^1^Department of Urology, Gazi University School of Medicine, 06500 Ankara, Turkey; ^2^Department of Medical Biology and Genetics, Gazi University School of Medicine, 06500 Ankara, Turkey; ^3^Department of Urology, Osmangazi University School of Medicine, 26040 Eskisehir, Turkey

## Abstract

*Aim*. To determine the impact of gene polymorphisms on detrusor contraction-relaxation harmony in children with lower urinary tract symptoms (LUTS). *Materials and Methods*. Toilet trained children older than 5 years of age with LUTS and normal neurological examination underwent videourodynamic study. The control group was composed of age matched children with no voiding complaints. The study group who filled out the voiding dysfunction symptom score before and after the treatment received standard oxybutynin treatment and was reevaluated 1 year after treatment. Genomic DNA was isolated from all patients and subjected to PCR for amplification. Genotyping of ARGHEF10, ROCK2, ADRB3, and CYP3A4 was carried out with Polymerase Chain Reaction- Restriction Fragment Length Polymorphism (PCR-RFLP) method. *Results*. 34 (45%) and 42 (55%) patients were enrolled in the study and control group, respectively. ARGEF10 GG, ADRB3 TC, and CYP3A4 AG genotype patients displayed insignificant difference between pre- and posttreatment voiding dysfunction symptom score and bladder volumes. *Conclusions*. The polymorphism of genes in the cholinergic pathway did not significantly differ clinical parameters. On the other hand, polymorphic patients in the adrenergic pathway seemed to suffer from clinical disappointment. For this reason, we think that the neglected adrenergic pathway could be a new therapeutic target for the treatment of anticholinergic resistant LUTS in children.

## 1. Introduction

Lower urinary tract symptoms (LUTS) are the most commonly encountered voiding dysfunction in children and are generally seen between 5 and 7 years of age [1]. Delay in the maturation of cortical inhibition of detrusor contractions is considered among the pathophysiological theories [2]. Treatment of LUTS was aimed only at the inhibition of muscarinic receptors in order to relax the detrusor muscle and antimuscarinics have been prescribed since the 1970s [3].

Stimulation of M3 receptor in detrusor muscle by acetylcholine results in activation of RhoA pathway leading to contraction and actin cytoskeletal reorganization [4]. There are 20 genes encoding Rho family in different biochemical pathways of human genome. Among these, certain polymorphisms are described in genes encoding ARHGEF10 and ROCK proteins. After the analysis of these genes, the polymorphism of ARHGEF10 resulted in a new binding region of Sp1 transcription factor and it was shown that expression of ARGHEF10 at transcriptional level might increase. In addition, the polymorphism described in ROCK gene was found to be in the dimerization region of ROCK protein close to the binding region of RhoA. This change was thought to be responsible for the diversity of the ROCK2 protein dimerization [5].

On the other hand, activation of adrenergic pathway in detrusor muscle through *β*-adrenergic receptors (AR) results in relaxation of detrusor muscle leading to the deposition of urine in bladder [6]. AR have 3 subtypes as *β*_1_,  *β*_2_, and *β*_3_ [6]. Among these, *β*_3_ AR mRNA is highly expressed in detrusor muscle [7, 8]. The most efficient relaxation is achieved through *β*_3_ AR agonists [9]. For this reason, *β*_3_ AR encoded by ADRB3 gene is thought to be mainstay of detrusor relaxation in human bladder and it might be considered as a new therapeutic target in the treatment of OAB [10–12].

Oxybutynin is mainly metabolized by P450-bound microsomal enzyme system in liver [13]. The cytochromosomal enzyme family responsible for the oxidation, reduction, and peroxidation of both oxybutynin and propiverine is CYP3A4 [13–15]. Enzyme activity level might display differences among people and races. In addition, there is a significant toxicity risk among people with low enzyme activity level [16]. The first variant described in this gene leading to the diminished enzyme activity is CYP3A41B [16]. For these reasons, it is speculated that the polymorphism of this enzyme family might cause a diversity in the metabolism of oxybutynin and diminish the efficacy of the medical treatment.

In our study, we aimed to determine the polymorphisms of the genes participating in detrusor contraction-relaxation harmony in kids with OAB and evaluate the impact of these polymorphisms on the clinical response to the medical treatment.

## 2. Materials and Methods

### 2.1. Study Population

Toilet trained children older than 5 years of age with urgency, frequency, urge incontinence, and normal neurological examination underwent videourodynamic study. Patients with vesicoureteral reflux, dysfunctional voiding pattern, constipation, and residual urine were excluded from the study. The control group was composed of age matched children with no voiding complaints who received treatment in our pediatric urology unit due to other urological pathologies.

In urodynamic evaluation before the treatment, bladder capacity, peak detrusor pressure, voiding pattern, and uroflowmetric parameters were recorded. All patients filled out the voiding dysfunction symptom score (VDSS) [17] before and after the treatment. VDSS is originally developed and validated by Akbal et al. [17]. It is one of the most widely used scales for assessing dysfunctional voiding and consists of 14 questions in total, 13 related to lower urinary tract symptoms and one related to quality of life. Questions from one to four assess enuresis. Four questions (numbers 5, 10, 11, and 12) gather data on filling phase symptoms and five questions (numbers 6–9 and 13) gather data on voiding symptoms. The study group received standard oxybutynin (0.3 mg/kg) treatment and was reevaluated with only VDSS and uroflowmetric analysis at 1 year after treatment because we were not allowed to perform a second urodynamic study after the treatment. Our patients were checked 3 months and 1 year after the prescription of the standard anticholinergic therapy. Patients with VDSS ≤8 and >8 were considered as responders and nonresponders, respectively. The posttreatment data was also assessed according to filling phase and voiding phase symptoms. This study was approved by the Committee of Ethics of the Gazi University and written informed consent was obtained from all of the participants and their parents.

### 2.2. Genotyping Analysis

Genomic DNA was collected from peripheral blood samples using the Heliosis Kit (Metis Biotechnology, Ankara, Turkey), following the manufacturer's instructions. ARGEF10, ROCK2, ADRB3, and CYP3A4 polymorphisms were genotyped by Polymerase Chain Reaction-Restriction Fragment Length Polymorphism (PCR-RFLP) methods. Amplifications were performed with Mastercycler gradient (Eppendorf, Hamburg, Germany) thermal cycler. The gene-specific primer sequences, PCR products, and restriction endonucleases (REs) are shown in Table 1. Amplifications of all four genes were performed in a final volume of 50 *μ*L, containing 3 *μ*L genomic DNA, 1.5 mM MgCl_2_, 0.2 mM dNTP, 50 pmol/mL of each primer, and 1.0 U/mL Taq DNA polymerase. Thermal cycling conditions were settled as 94°C for 30 seconds, 62°C for 60 seconds for 30 cycles, and then 72°C for 1 minute. After the incubation time, the fragments were separated on a 2%–4% agarose gel that was stained with ethidium bromide and visualized by Gel Logic 100 image system (Kodak, Rochester, NY).

### 2.3. Statistical Analysis

Differences between the means of two continuous variables were evaluated by Student's *t*-test, Mann-Whitney *U* test, and Fisher's exact test. Using the *χ* ^2^ test, polymorphisms were tested for deviation from Hardy-Weinberg equilibrium (HWE). The relative association between patients and controls for genotype and allele frequencies was assessed by Pearson's *χ* ^2^ test. The corresponding odds ratios (ORs) and confidence intervals (95% CIs) were calculated with SPSS version 15.0. Multivariate unconditional logistic regression analysis with adjustment for clinical and biochemical characteristics and genotype distribution was performed to calculate adjusted ORs and 95% CIs. All *p* values are two sided; *p* values <0.05 were considered statistically significant.

## 3. Results

34 (45%) and 42 (55%) patients were enrolled in the study and control group, respectively. The demographic and clinical characteristics of responder and nonresponder patients treated with oxybutynin are summarized in Table 2. VDSS before the treatment in responder and nonresponder group were 14.44 (10–25) and 19.89 (8–28), respectively (*p* = 0.063).

On the other hand, VDSS after the treatment was significantly lower in the responder group 2.28 (0–8) when compared with the nonresponder group 14.11 (8–20) (*p* < 0.001). When we evaluated our patients according to filling and voiding phase symptoms after treatment, responders had significantly less bothering symptoms in both phases when compared to the nonresponder group (*p* = 0.05; *p* = 0.045). In addition, there was no significant difference in terms of uroflowmetric bladder volume before and after the treatment between the responder and nonresponder groups (*p* = 0.48; *p* = 0.7).

When patients in the treatment arm are grouped according to the allelic types, only ARGEF10 GG, ADRB3 TC, and CYP3A4 AG patients displayed insignificant difference between pre- and posttreatment VDSS and bladder volumes (Table 3).

All four SNP genotype frequencies in both study and control group were in agreement with Hardy-Weinberg equilibrium (*p* > 0.05).

As for ARGEF10 (rs4376531) genotypes, responder group (*n* = 26) of the 34 patients, 20 patients had the CC genotype (76.9%), 4 patients had the CG genotype (15.4%), and 2 patients had the GG genotype. Of the nonresponder group (*n* = 8), 6 cases were type CC (75%) and 2 cases were CG (25%). Of the 42 healthy control cases, 29 (69%) cases were type CC, 12 (28.6%) cases were CG (2.4%), and only one case was type GG. The frequencies of C alleles were 85.3% in both responder and nonresponder patients and 83.3% in control group. The frequencies of G alleles were 16.7% in both responder and nonresponder patients and in control group. We found no statistically significant difference in the genotype and allele frequencies between the patients and controls.

As for ROCK2 (rs2230774) genotypes, of the responder patients, 12 patients had the AA genotype (48%), 9 patients had the AC genotype (36%), and 4 patients had the CC genotype (16%). Of the nonresponder patients, 3 patients had the AA genotype (33.3%), 3 patients had the AC genotype (33.3%), and 3 patients had the CC genotype (33.3%). Of the healthy control cases, 13 cases were type AA (31.7%), 17 cases were AC (41.5%), and 11 cases were type CC (26.8%). The frequencies of A alleles were 61.8% in all patients and 52.4% in control group. The frequencies of C alleles were 38.2% in all patients and 47.6% in control group. Genotype and allele frequencies were not significantly different between the patients and the control group (*p* > 0.05).

As for ADRB3 rs4994 T/C genotypes, responder group (*n* = 26) of the 34 patients, 23 patients had the TT genotype (88.5%) and 3 patients had the TC genotype (11.5%). Of the nonresponder group (*n* = 8), 7 cases were type TT (87.5%) and only one case was TC (12.5%). Of the 42 healthy control cases, 37 (88.1%) cases were type TT and 5 (11.9%) cases were TC. CC genotype was observed neither in patients nor in healthy controls group. The frequencies of T alleles were 94.1% in both responder and nonresponder patients and 94% in control group. The frequencies of C alleles were 5.92% in both responder and nonresponder patients and 6% in control group. We found no statistically significant difference in the genotype and allele frequencies between the patients and controls.

As for CYP3A4^*∗*^1B (rs2740574) genotypes, of the responder patients, 23 patients had the AA genotype (92%) and 2 patients had the AG genotype (8%); of the nonresponder patients, 8 patients had the AA genotype (88.9%) and only one patient had the AG genotype (11.1%). Of the healthy control cases, 36 cases were type AA (87.8%) and 5 cases were AG (12.2%). The frequencies of A alleles were 95.6% in all patients and 93.9% in control group. The frequencies of G alleles were 4.4% in all patients and 6.1% in control group. Genotype and allele frequencies were not significantly different between the patients and the control group (*p* > 0.05).

## 4. Discussion

Humans are believed to carry over a million distinct single nucleotide polymorphisms (SNPs). The determination of SNPs is a new means to study the etiology of polygenetic disorders with complex inheritance patterns, like OAB. Polymorphism of ARGEF10 gene was studied in numerous pathologies and special focus was directed on systemic hypertension because of the aforementioned effect of this gene on smooth muscle contraction. Matsushita et al. [18] compared 2775 patients with 2839 control cases in terms of thrombotic attack and evaluated four different SNPs related with ARGHEF10. They found that the transcriptional activity of ARGHEF10 was affected by differentiated Sp1 binding affinity due to rs4376531. As a result, they concluded that rs4376531 enhanced RhoA activity. Particularly, they declared that CC and CG alleles increased the risk of ischemia because they increased smooth muscle tonus in the tissue (*p* = 0.033). In our study, although we could not detect significant impact of our genotypes on VDSS when evaluated together, CC and CG allele of ARGHEF10 displayed significant impact on VDSS. In contradiction to Matsushita et al. [18], these patients responded well to standard anticholinergic treatment and their VDSS decreased. This contradiction could be the result of different ethnic group of patients included in these studies and it can also rise from the fact that level of ARGHEF10 was studied from serum instead of the plasma like in our study. In addition, because we had few patients with GG genotype in the study group, this could also support the statistical insignificance.

ROCK2 has important functions in smooth muscle contraction. Kandabashi et al. [19] studied a hypertension model in rats and found that upregulation of ROCK2 was related with coronary spasm. On the other hand, inhibition of ROCK2 by certain medical agents led to normotension [20, 21]. Peterson et al. [22] evaluated allelic variant of ROCK2 in women with preeclempsia and proposed that ROCK2 could be a functional target for the regulation of preeclempsia. We detected two homozygote and one heterozygote genotypes for ROCK2 in our study. Among these, AC was found to be the most abundant. When ROCK2 genotypes were evaluated separately, VDSS decreased and bladder capacity increased significantly in both alleles. This result lets us think that polymorphism of ROCK2 gene has no impact on the response to anticholinergic treatment. Furthermore, ARHGEF polymorphisms might activate binding of RHOA to ROCK2 and allow ROCK2 inhibitory phosphorylation of Thr696.

Oxybutynin has been prescribed enormously and displayed significant side-effects due to the nonselective anticholinergic activity. After that, intravesical application of the medication has begun in order to bypass the side-effects [23]. Due to the invasive nature of the application, more selective medical treatment options were evaluated. Goessl et al. [24] declared 33% cure and 13% side-effect rate with tolterodine treatment in children with OAB, respectively. Yucel et al. [25] found that tolterodine treatment increased the efficacy of the pharmacotherapy in oxybutynin intolerant children. Because of the dissatisfactory results obtained from the studies in cholinergic pathway, researchers head towards the disregarded adrenergic pathway. Nomiya and Yamaguchi [26] found the dominance of *β*_3_ AR subtype in the bladder. In animal studies, it was found that certain *β*_3_ AR agonists (FK175, CL316243) could significantly improve bladder capacity and prolong voiding intervals without interference of voiding pressure [27, 28]. No significant cardiac side-effects were detected in these studies. On the other hand, other *β*_3_ AR agonists (BRL37344A and CL316243) could not display significant relaxative effects on human detrusor muscle cell in contradiction to rat detrusor [29].

The frequency of the polymorphism that we studied (ADRB3 Trp64Arg (rs4994) T/C) was found to be 20% and 40% in Japanese and Alaskan Eskimos, respectively [29]. These results show that the aforementioned polymorphism is commonly found in the community. In these studies, it was declared that polymorphic receptor led to the diminished cAMP activity when compared with wild-type counterpart. Therefore, this molecular event might result in less relaxation of the detrusor muscle. In our study, we also found that children with polymorphic allele had significantly less bladder capacity with higher VDSS. Honda et al. [30] evaluated women with idiopathic OAB and proposed ADRB3 Trp64Arg (rs4994) T/C polymorphism as the probable molecular explanation of this disease. They found 47% and 22.8% polymorphic patients in the study and control group, respectively (*p* < 0.05). We could not reach similar distributional significance between the study and control groups probably due to small sample size.

Oxybutynin is excreted through metabolism of the drug to N-deethyloxybutynin by liver [31]. Cytochrome P450-bound isoenzyme is mainly responsible for this oxidation process. Yaïch et al. [31] showed that CYP3A4 was responsible for the metabolism of oxybutynin and propiverine. Wang et al. studied CYP3A4-22 polymorphism in patients consuming statin and found that patients without T allele had to consume 1.7–5 times less statin than the patients with T allele to reach the same plasma lipid level. This study clearly shows that polymorphic patients can be considered as hypermetabolizers for statin drugs [32]. In a study by Muller et al. [33] it was found that hyper- and hypometabolizers of propiverine displayed similar drug pharmacokinetics and side-effects. In our study, we tried to determine the impact of the polymorphism of drug metabolism on clinical parameters in our patients who received oxybutynin. There was no significant difference in the distribution of allele frequency between the study and the control group. These findings let us think that metabolism of oxybutynin was similar in both groups. On the other hand, when the impact of different alleles in CYP3A4 on clinical parameters was evaluated, children with AA allele responded better than the AG allele to standard oxybutynin regimen. Although this clinical scenario could be affected by numerous parameters, it might be speculated that G allele could result in enhancement of the drug metabolism. Thus, half-life of the drug could be shortened and sufficient clinical improvement could not be achieved. On the other hand, this assumption should be supported with larger sample size of patients to reach a consistent conclusion.

## 5. Conclusion

Anticholinergic medication is contemporarily considered as the first treatment of choice for children with OAB. Although this method is very efficient, a significant portion of the patients suffer from unsatisfactory clinical improvement and side-effects. We studied certain genes in the molecular pathway of detrusor contraction-relaxation harmony and drug metabolism. We did not detect significant impact of these polymorphisms on clinical parameters as a whole probably due to small sample size. The polymorphism of genes in the cholinergic pathway could not significantly differ clinical parameters. On the other hand, polymorphic patients in the adrenergic pathway seemed to suffer from clinical disappointment. For this reason, we think that the neglected adrenergic pathway could be a new therapeutic target for the treatment of anticholinergic resistant OAB in children.

## Figures and Tables

**Table 1:** The gene-specific primer sequences, PCR products, and restriction endonucleases (REs).

Polymorphisms	Primers	PCR products (bp)	REs	Alleles (bp)
ARHGEF10 (rs4376531) C>G	F: 5′CACCACTAAACATGCCCTAT3′ R: 5′TAACAACGAAAAGTAGTAGC3′	369	*Psu*I	C allele: 369 G allele: 153, 216

ROCK2 (rs2230774) A>C	F: 5′AGTGACTCTCCATCTTGTAGAGAAG3′ R: 5′AGCCATGTAGAAGTCCTCTTTG3′	274	*MboII *	A allele: 274 C allele: 34, 240

ADRB3 (rs4994) T>C	F: 5′CCAGTGGGCTGCCAGGGG3′ R: 5′GCCAGTGGCGCCCAACGG3′	248	*Mva*I	T allele: 97, 62, 61, 15, 13 C allele: 158, 62, 15,13

CYP3A4 (rs2740574) A>G	F: 5′GGAATGAGGACAGCCATAGAGACA AGGGGA3′ R: 5′CCTTTCAGCTCTGTGTTGCTCTTTGCTG3′	385	*Mbo*II	A allele: 175, 169, 41 G allele: 210, 175

**Table 2:** Demographic and clinical features of responder and nonresponder patients.

Variable	Responder	Nonresponder	*p* value
Gender *n* (%)			
Female	18 (78,3)	5 (21,7)	0,37
Male	7 (73,4)	4 (36,6)	
Age mean (Min–Max)	8,64 (5–16)	11,44 (5–17)	0,11
Renal scar *n* (%)			
−	20 (29,9)	8 (11,9)	0,55
+	5 (62,5)	1 (12,5)	
VDSS mean (Min–Max)			
Before treatment	14,44 (10–25)	19,89 (8–28)	0,063
After treatment	2,28 (0–8)	14,11 (8–20)	**<0,001**
Filling phase symptoms mean (Min–Max)			
Before treatment	4,85 (4–6)	5,1 (3–6)	0,084
After treatment	1,22 (0–3)	2,47 (2–4)	**0,05**
Voiding phase symptoms mean (Min–Max)			
Before treatment	8,23 (5–10)	8,46 (6–9)	0,46
After treatment	2,34 (0–4)	3,48 (3–8)	**0,045**
Bladder volume (mL) mean (Min–Max)			
Before treatment	167,08 (80–350)	214,67 (100–521)	0,48
After treatment	307,84 (120–600)	291,78 (130–526)	0,7

**Table 3:** Pre- and posttreatment VDSS and bladder volumes according to ARGEF10, ADRB3, ROCK, and CYP3A4 allelic types.

Gene allele	Pretreatment VDSS mean ± SD	Posttreatment VDSS mean ± SD	*p*	Pretreatment bladder volume (mL) mean ± SD	Posttreatment bladder volume (mL) mean ± SD	*p*
ARGEF10						
CC	7,72 ± 9,26	2,50 ± 5,25	**<0,001**	183,50 ± 63,92	312,86 ± 122,75	**<0,001**
CG	6,67 ± 10,80	2,44 ± 5,93	**0,026**	178,29 ± 156,13	260,43 ± 141,97	**0,030**
GG	9,33 ± 10,07	1,67 ± 2,89	0,343	125 ± 35,36	275 ± 106,07	0,205
ADRB3						
TT	7,83 ± 9,47	2,41 ± 5,22	**<0,001**	179,25 ± 89,08	308,84 ± 130,58	**<0,001**
TC	5,82 ± 10,33	2,73 ± 6,07	0,111	180 ± 67,45	250 ± 55,23	0,199
ROCK						
AC	6,76 ± 9,82	1,83 ± 4,86	**0,001**	211,23 ± 121,29	304,15 ± 154,68	**0,020**
AA	8,11 ± 8,64	2,57 ± 4,94	**0,001**	178,20 ± 57,98	325,57 ± 116,36	**<0,001**
CC	7,89 ± 10,9	3,28 ± 6,59	**0,016**	135,22 ± 35,11	255,56 ± 80,33	**0,001**
CYP3A4						
AA	7,78 ± 9,52	2,52 ± 5,34	**<0,001**	172,97 ± 83,53	295,41 ± 126,85	**<0,001**
AG	5,5 ± 10,31	1,88 ± 5,30	0,280	251,67 ± 91,88	363 ± 78,08	0,075
